# Medical Society of the County of Erie

**Published:** 1904-05

**Authors:** Franklin C. Gram


### Medical Society of the County of Erie.

*Special Meeting held April 9, 1904.*

REPORTED BY FRANKLIN C. GRAM, M. D., Secretary.

A special meeting of the Medical Society of the County of Erie was held at the rooms of the Buffalo Society of Natural Sciences in the Public Library Building, Saturday, April 9, 1904, at 4 p. m., for the purpose of taking action on the report of the joint committee of conference, consolidating the Medical Society of the State of New York and the New York State Medical Association.

The president, Dr. William C. Krauss of Buffalo, took the chair at 4.15 p. m., and, in the absence of the regular secretary, appointed Dr. William G. Ring secretary pro tempore. The president then stated the object of the meeting as recited above. He said it is held pursuant to a notice from the secretary of the Medical Society of the State of New York, which he directed the secretary to read, and which was done.

Dr. William Warren Potter then offered the following preamble and resolutions:

Whereas, Duly appointed committees of conference on the part of the Medical Society of the State of New York and of the New York State Medical Association have agreed upon a plan of union of these two bodies and of their constituent societies and associations; and,

Whereas, The two original contracting parties to the conference—namely, the society and the association, have unanimously ratified and confirmed the report of the committees; therefore, be it

*Resolved,* That the Medical Society of the County of Erie in special session assembled this 9th day of April, 1904, hereby approves the action of the said committees and joins in accepting the conditions of the aforesaid agreement so far as they may apply to this society; and, furthermore, this society agrees on its part to do whatever may be legally necessary to complete the consolidation and make it effective, and the Medical Society of the County of Erie hereby waives notification of an application to court for an order consolidating said corporations pursuant to the terms of said agreement, and hereby consents to the entry of such an order without notice; and be it further

*Resolved,* That the secretary of this meeting be and he is hereby authorised and directed to send a copy of these resolutions, duly certified by the president and secretary of the meeting, to the secretary of the Medical Society of the State of New York, and to execute and deliver any and all waivers of notice of an application for such order as the court may require.

In moving their adoption Dr. Potter said that, in view of the probability that all present were familiar with the action already taken by the two state medical bodies at interest, it would be quite unnecessary for him to enter into a detailed explanation of the subject. A schism that had existed for twenty-two years was about to pass; only a few formal acts being necessary to wipe it out.

Dr. DeLancey Rochester seconded the motion of Dr. Potter in a brief but well expressed speech, during the course of which he stated that he had been an advocate of the consolidation movement in and out of season, among the members of both bodies, sometimes to the irritation of his association colleagues. He was glad to note the near accomplishment of this important procedure, which should be gratifying to every member of the medical profession in the state of New York.

Further remarks in the same general spirit were made by Drs. J. B. Coakley, W. C. Callanan and others. The motion was then unanimously adopted and the meeting was adjourned.

